# The Effect of Infectious Disease Threat on Malevolent Creativity

**DOI:** 10.3390/jintelligence10040111

**Published:** 2022-11-21

**Authors:** Mingzhe Zhao, Ke Zhang, Xiumin Du

**Affiliations:** College of Education, Hebei University, Baoding 071002, China

**Keywords:** disease threat, behavioral immune system, malevolent creativity

## Abstract

The behavioral immune system (BIS), which evolved to protect humans from infectious disease threats, prompts people to be sensitive to disease-connoting cues. A common denominator of many disease-connoting cues is benign physical abnormalities, such as birthmarks and obesity. Previous studies found that among people whose BIS was activated (e.g., people who were exposed to situational disease prime or chronically concerned about disease threat), disease-connoting cues could make people feel threatened by infectious disease and induce their malevolence. Malevolence is a necessary feature of malevolent creativity (MC), which is defined as creativity that deliberately leads to harmful or immoral results. According to the motivated focus account of creativity, a threat could promote creativity when creativity is relevant to the threat. Thus, infectious disease threats might increase malevolent creativity. However, whether infectious disease threats could influence MC is unknown. Therefore, the current study aims to explore the effect of infectious disease threat on MC by two disease-connoting cues (birthmark, obesity). In Study 1 (*n* = 174), a 2 (threat prime: infectious disease, natural disaster) × 2 (disease-connoting cue: birthmarked face, normal face) between-subjects design was used. Participants were asked to complete a malevolent creativity task (MCT). In Study 2 (*n* = 131), we used a perceived vulnerability to disease scale (PVD) to assess people’s dispositional tendencies of concerns about disease and selected high as well as low PVD participants. A 2 (PVD: high, low) × 2 (disease-connoting cue: obese, average-weight) between-subjects design was used. Participants were asked to complete the negotiation task to assess their MC. The results of Study 1 showed that, compared with participants in the normal face condition, participants in the birthmarked face condition showed higher MC fluency and total MC when they were exposed to situational disease prime. Compared with the natural disaster prime group, the infectious disease prime group showed higher MC fluency and total MC when they were provoked by a birthmark person. The results of Study 2 showed that, compared with the average-weight condition, the obese condition led to higher MC fluency and originality among high PVD participants. Compared with low PVD participants, high PVD participants showed higher MC fluency and originality when they negotiated with an obese person. Our studies suggest that among people whose BIS is situationally or chronically activated, birthmarks and obesity could increase MC, and people’s malevolent creativity might be induced by disease-connoting cues during the pandemic.

## 1. Introduction

Perhaps, in the evolutionary history of humans, no other element has shaped our behavior more than infectious diseases. Different from other kinds of threats, infectious disease threats are invisible to the naked eye and can transmit from person to person. Besides, they often elicit an emotion: disgust (Curtis et al. 2004). In the 21st century, with highly developed medical technology, viruses like Ebola are still causing huge losses of human life (World Health Organization. Regional Office for the Western Pacific 2016). In particular, as of 27 September 2022, COVID-19, an epidemic that broke out in 2019, has infected more than 612 million people and led to the death of more than 6.5 million people (World Health Organization 2022).

To deal with infectious disease threats, humans evolved a behavioral immune system (BIS). It consists of affective (e.g., disgust; Curtis et al. 2004), cognitive (e.g., higher attention; Ackerman et al. 2009), and behavioral (e.g., avoidance behavior; Miller and Maner 2011) processes that facilitate the detection and avoidance of disease-carrying individuals (Schaller and Park 2011). The function of BIS follows two principles. Firstly, BIS follows the “Smoke Detector Principle”—it prompts people to be sensitive to cues displayed by others that are heuristically associated with the presence of disease (Nesse 2005). A common denominator of many disease-connoting cues is benign physical abnormalities, such as obesity, birthmarks, and strabismus (Ackerman et al. 2009; Lund and Miller 2014; Miller and Maner 2011; Park et al. 2007). Secondly, the activation of BIS usually has costs (e.g., avoiding potential disease carriers may result in missed chances of social interaction). Because not all situations afford identical levels of infectious disease threat, and people differ in their sensitivity to an infectious disease threat, the BIS follows the “Functional Flexibility Principle”—the costs and benefits of activating the BIS need to be balanced (Schaller and Park 2011; Schaller et al. 2007).

Among people whose BIS is situationally or chronically activated (e.g., people primed with an infectious disease or people high in perceived vulnerability to disease scale (PVD; Duncan et al. 2009)), disease-connoting cues (e.g., birthmark, obesity) in the environment could make people feel threatened by infectious disease and influence their cognitions. Recent studies found that participants in the disease prime group would pay more attention to birthmarked faces as well as people with strabismus (Ackerman et al. 2009), and set a lenient threshold for categorizing targets as obese as well as reporting that they had previously seen obese people (Miller and Maner 2012). Similar results were also found among high PVD participants (Miller and Maner 2012). Besides, previous studies found that chronically feeling vulnerable to disease (e.g., high in PVD or disgust sensitivity) resulted in perceiving unfamiliar people as less similar to oneself (Mentser and Nussinson 2020) and higher prejudice towards obese individuals (Magallares et al. 2015; Park et al. 2007).

Although researchers have examined the effects of disease threats on some cognitive processes, little research has examined the effect of disease threats on creativity—one of the most important cognitive functions of humans. It is defined as the development of ideas, products, or problem solutions that are both original and useful (Runco 2004; Runco and Jaeger 2012). We considered that infectious disease threats might be involved with creativity. According to the motivated focus account of creativity (De Dreu and Nijstad 2008), threats can increase people’s motivation to deal with the threatening situation. This heightened motivation drives them to mobilize cognitive resources to process threat-relevant information (Reinecke et al. 2009) and search for effective solutions to cope with the threat at hand. Thus, a threat could promote people’s creativity. Recent studies found that the violence threat (Cheng et al. 2018) and social threat (De Dreu and Nijstad 2008) could promote people’s creativity when their creativity was relevant to the threats at hand. Thus, as a kind of threat, infectious disease threats might increase the idea generation of creativity.

Creativity includes malevolent creativity (MC), benevolent creativity (BC), and neutral creativity (NC; Baas et al. 2019). MC is typically defined as the creative ideas which are generated to purposefully inflict material, mental, or physical harm on others (Cropley et al. 2014). It includes three dimensions: fluency, originality, and malevolence (Perchtold-Stefan et al. 2021). Malevolence might be induced due to the presence of infectious disease threats. Previous studies found that perceived vulnerability to COVID-19 was significantly positively correlated to both aggression and anger towards individuals who posed a disease threat (Loustau 2021), and both aggression and anger were positively correlated to malevolent creativity (Cheng et al. 2021; Hao et al. 2020; Harris and Reiter-Palmon 2015; Lee and Dow 2011; Perchtold-Stefan et al. 2021). People, who are situationally exposed to disease prime or chronically concerned about disease threat (e.g., high PVD trait), display prejudice and exclusion towards individuals with disease-connoting cues (Huang et al. 2011; Lund and Miller 2014; Magallares et al. 2015; Makhanova et al. 2015; Park et al. 2007). According to the motivated focus account of creativity (De Dreu and Nijstad 2008) and the induced malevolence, infectious disease threats might drive people’s cognitive resources to malevolently deal with problems. Thus, infectious disease threats might increase malevolent creativity rather than other kinds of creativity (e.g., BC, NC). However, whether infectious disease threats could influence MC is unknown.

The current study aims to explore the effect of infectious disease threat on MC by two disease-connoting cues (birthmark, obesity). According to the “Functional Flexibility Principle” (Schaller and Park 2011; Schaller et al. 2007), compared with natural disaster prime that is often used as a control in BIS activating experiments, when people are exposed to infectious disease prime, disease-connoting cues could make them feel threatened by infectious diseases. Besides, compared with low PVD people, disease-connoting cues could make high PVD people feel threatened by infectious diseases. Therefore, across two studies, we separately explored whether situationally (Study 1) and chronically (Study 2) activated BIS would lead to higher MC towards disease-connoting cues (birthmark, obesity). Malevolent creativity task (MCT) and negotiation task are two classical tasks for measuring MC. Whereas the former task is malevolent-oriented (e.g., participants are told to come up with original ways of revenge; Hao et al. 2020; Perchtold-Stefan et al. 2021; Xu et al. 2021), the latter task is not. In the negotiation task, the generated creative ideas could be malevolent, benevolent, or neutral (Baas et al. 2019). In this article, we assessed MC through these two different tasks. In Study 1, we tested whether birthmarks could influence MC when participants were exposed to situational infectious disease prime or natural disaster prime. In this study, participants were asked to complete MCT, in which they were provoked by the person with a birthmark on his/her face or not and instructed to generate as many original ideas as possible to take revenge on the birthmark person or the normal person. Compared with natural disaster prime, we predicted that infectious disease prime might induce higher levels of MC when participants were provoked by a birthmark person. Compared with a normal face, we predicted that a birthmarked face might induce a higher level of MC when participants were exposed to infectious disease prime. In Study 2, we tested whether obesity could influence MC among high and low PVD participants. In this study, high and low PVD participants were selected and told that they would negotiate with an obese person or an average-weight person. They were asked to generate tactics for the upcoming negotiation. Compared with a low PVD trait, we predicted that a high PVD trait might induce a higher level of MC when participants negotiated with an obese person. Compared with an average-weight person, we predicted that an obese person might induce higher MC among high PVD participants.

## 2. Study 1

### 2.1. Experimental Design

A 2 (threat prime: infectious disease, natural disaster) × 2 (disease-connoting cue: birthmarked face, normal face) between-subjects design was used. The dependent variables were indicators of malevolent creativity: fluency, originality, malevolence, and total malevolent creativity.

### 2.2. Participants

In total, 174 Chinese college students were recruited. Four participants who generated zero ideas on the MCT were excluded from the analyses. Two participants were identified as outliers (more than three standard deviations from the mean for malevolent creativity indicators) and removed from further analyses. The final sample included 168 participants (aged 17 to 27, *M*_age_ = 19.73, *SD*_age_ = 2.04). Among them, 51 (30.4%) were males and the remaining 117 (69.6%) were females.

### 2.3. Materials

#### 2.3.1. Materials of Threat Prime and Manipulation Checks

Two video clips were used to prime threats. The infectious disease video (03:01 min), which depicted the rapid spread of the influenza virus among humans, was edited from the movie *the flu*. The natural disaster video (03:03 min), which depicted the warning signs of a tsunami and the scenes as it came, was edited from the movie *Lo imposible*. Manipulation checks of threat prime were assessed with 13 items (1 = not at all, 4 = not sure, 7 = very much): 6 items for emotions (“At this very moment, I feel happy/sad/tense/surprise/disgust/fear”; Ji et al. 2019; Stevenson et al. 2011), 1 item for perceived threat level (“I thought the video threatening”; Cheng 2017), 2 items for content relevance (“I thought the video depicted content of infectious disease/natural disaster”; Li 2018; Nussinson et al. 2018), 4 items for threat concerns (“I felt concerned about infectious disease/natural disaster because of this video clip”; “I was motivated by this video to prevent the harmful effect of infectious disease/natural disaster”; Brown and Sacco 2020; Cheng 2017).

#### 2.3.2. Malevolent Creativity Task

Malevolent creativity problem-solving ability was assessed by the malevolent creativity task (MCT). The scenario of MCT, which we used in Study 1, was developed by Yang (2016) according to the purport of previous malevolent creativity tasks (Hao et al. 2020; Perchtold-Stefan et al. 2021, 2022; Xu et al. 2021). Specifically, participants face the following scenario: “You and *Xiao He* (a Chinese name) are roommates. You’re a light sleeper and have trouble falling back asleep once you wake up. However, *Xiao He* often returns dormitory late, and the noise of washing always wakes you up. Therefore, you often suffer from sleeplessness and have no energy to do affairs during the day. You negotiate with *Xiao He* about this many times, but *Xiao He* doesn’t listen and still goes his/her own way”. Participants were told to imagine the scenario happening to them and picture it as vividly as possible. Then, participants were given 8 min to generate as many original ideas as possible to take revenge on *Xiao He* secretly (Perchtold-Stefan et al. 2021). Two trained psychological graduate students who were familiar with the field of creativity and were blind to the conditions and hypotheses coded the ideas generated over the MCT. The fluency index was calculated as the total number of non-identical ideas identified as at least slightly malevolent by both raters (88.8% of all ideas, ICC = 0.83; Perchtold-Stefan et al. 2021). The same two raters evaluated malevolent ideas’ originality (4-point scale, 1 = not original, 4 = very original, ICC = 0.73) and malevolence (4-point scale, 1 = slightly malevolent, 4 = highly malevolent, ICC = 0.72; Amabile 1982; Perchtold-Stefan et al. 2021). MC requires ideas to qualify as both, malevolent, and original. Therefore, the total malevolent creativity score was computed by summing up all malevolent ideas with at least moderately original (at least 2 on the rating of originality; Perchtold-Stefan et al. 2021). 

#### 2.3.3. Birthmarked Faces

Four faces (two birthmarked faces and two normal faces) were used. Firstly, one male and one female Asian face displaying neutral expressions and of similar age as our participants were chosen from the Chicago face database (Ma et al. 2015). We used these two faces as normal faces. Then, computer software was used to add a piece of sepia birthmark to the normal male face. The same birthmark was added to the normal female face. The locations of the male birthmark and the female birthmark are identical. 

#### 2.3.4. Questionnaires

The perceived vulnerability to disease scale (PVD) was used to assess chronic beliefs about personal susceptibility to infectious disease (Duncan et al. 2009). In this study, we adopted the Chinese version of PVD, which was used in the previous study (Wu et al. 2019). It consists of two subscales: germ aversion (GA) and perceived infectability (PI). Participants responded to 15 items on a 7-point scale from 1 (strongly disagree) to 7 (strongly agree). Higher scores indicate greater disease concerns. In Study 1, Cronbach’s α coefficient for PVD was 0.71.

The Malevolent Creativity Behavior Scale (MCBS) was used to assess participants’ individual MC potential (Hao et al. 2016). It contains 13 items and includes three dimensions: hurting people, lying, and playing tricks. Participants were asked to choose on a 5-point scale from 0 (never) to 4 (usually) according to the frequency of these behaviors in their own daily lives. Higher scores indicate greater MC potential. The Cronbach’s α coefficient for MCBS was 0.85.

The Runco Ideational Behavior Scale (RIBS) was used to assess participants’ individual creative potential (Runco and Acar 2012). The short version of RIBS was adopted in this study (Hao et al. 2020). It contains 19 items. Participants were asked to choose on a 5-point scale from 0 (never) to 4 (just about every day) according to the frequency of their thoughts in their daily lives. Higher scores indicate greater creative potential. The Cronbach’s α coefficient for RIBS was 0.82.

### 2.4. Procedure

**Figure 1:**
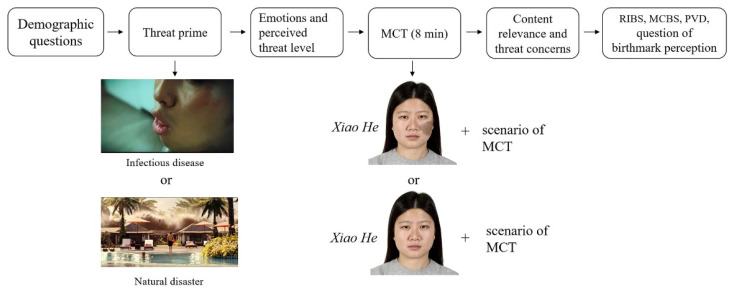
Procedure for female participants in Study 1.

We designed the procedure of Study 1 based on the experimental paradigm used in previous studies, in which participants were first primed with threats and then completed a cognitive task that may include disease-connoting cues (Ackerman et al. 2009; Lund and Miller 2014; Miller and Maner 2012). Specifically, in Study 1, participants first answered basic demographic questions. Then, they were randomly assigned to one of the threat prime groups and instructed to watch a video clip with headphones. After that, they answered their emotions and the threat level they perceived for manipulation checks. Then, they were randomly assigned to a birthmarked face or normal face condition. Based on the assignment, a birthmarked or normal face was presented with the scenario of MCT. The face presented on the screen was of the same sex as the participant and labeled “*Xiao He*”. Participants were asked to read the scenario. After that, they were given 8 min to generate as many original ideas as possible to take revenge on *Xiao He* secretly and type them into the text-entry box (the scenario and the face were presented on the screen until participants finished the task). After completing MCT, participants were instructed to recall the content of the video clip and answer questions to assess content relevance and threat concerns. Then, they completed RIBS, MCBS, and PVD questionnaires. In the end, participants in the birthmarked face condition orally and freely reported what they perceived the foreign matter on the face of *Xiao He* as. All the participants received CNY8 as their compensation. Figure 1 presents the procedure of Study 1.

### 2.5. Result

All the participants in the birthmarked face condition perceived the foreign matter on the face of *Xiao He* as a birthmark.

#### 2.5.1. Manipulation Checks of Threat Prime

For emotion ratings, the results of independent *t*-tests found that there was no significant difference between the two threat prime groups for happy, sad, tense, surprise, and fear ratings (*ps* ≥ 0.136). However, participants in the infectious disease prime group reported higher disgust than participants in the natural disaster prime group (*t*(154.58) = 7.92, *p* < 0.001, *d* = 1.22). For the perceived threat level, participants in both threat prime groups perceived the videos as equally threatening (*t*(166) = 0.97, *p* = 0.923, *d* = 0.02). For the ratings of content relevance and threat concerns, compared with participants in the natural disaster prime group, participants in the infectious disease prime group perceived the clip that they watched as more related to the content of disease (*t*(166) = 29.84, *p* < 0.001, *d* = 4.59) and reported higher disease concerns (*t*(135.13) = 26.48, *p* < 0.001, *d* = 4.12). On the contrary, compared with participants in the natural disaster prime group, participants in the infectious disease prime group perceived the clip that they watched as less related to the content of natural disaster (*t*(113.73) = −26.42, *p* < 0.001, *d* = −4.04) and reported lower natural disaster concerns (*t*(115.33) = −16.42, *p* < 0.001, *d* = −2.52). These results showed that our manipulation of threat prime was successful. Means and standard deviations of manipulation checks are reported in Table 1.

#### 2.5.2. Malevolent Creativity

Means and standard deviations of MC in Study 1 are presented in Table 2.

We submitted fluency, malevolence, originality, and total MC to separate 2 (threat prime: infectious disease, natural disaster) × 2 (disease-connoting cue: birthmarked face, normal face) ANOVAs with RIBS, MCBS, and PVD as covariates. 

**Figure 2:**
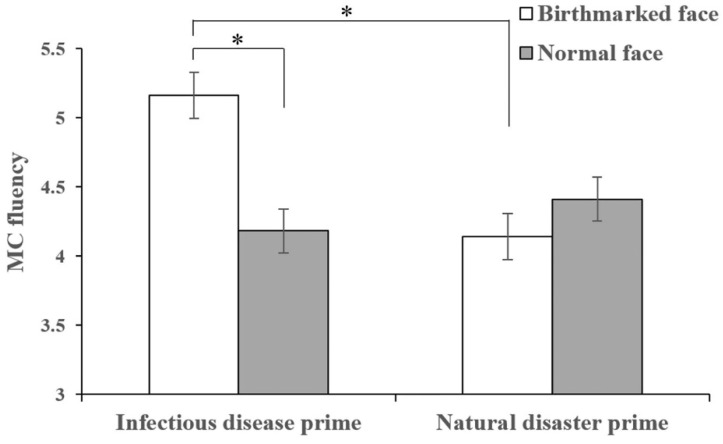
The influence of the interaction between threat prime and disease-connoting cue on MC fluency (displayed ± *SE*). * *p* < 0.05.

For the MC fluency, results revealed no main effects of threat prime (*F*(1, 161) = 1.31, *p* = 0.254, *η*^2^*_p_* < 0.01) and disease-connoting cue (*F*(1, 161) = 1.68, *p* = 0.196, *η*^2^*_p_* = 0.01), but did reveal a significant interaction between threat prime and disease-connoting cue (*F*(1, 161) = 4.80, *p* = 0.030, *η*^2^*_p_* = 0.03, see Figure 2). As expected given our results, for those in the infectious disease prime group, there was a significant effect of disease-connoting cue (*F*(1, 161) = 6.15, *p* = 0.014, *η*^2^*_p_* = 0.04), with greater MC fluency in the birthmarked face condition than in the normal face condition. For those in the natural disaster prime group, there was no significant effect of disease-connoting cue (*F*(1, 161) = 0.39, *p* = 0.531, *η*^2^*_p_* < 0.01). Besides, for those in the birthmarked face condition, there was a significant effect of threat prime (*F*(1, 161) = 5.73, *p* = 0.018, *η*^2^*_p_* = 0.03), with greater MC fluency in the infectious disease prime group than in the natural disaster prime group. However, for those in the normal face condition, there was no significant effect of the threat prime (*F*(1, 161) = 0.53, *p* = 0.469, *η*^2^*_p_* < 0.01).

For the results of malevolence and originality, we both found a main effect of threat prime (malevolence *F*(1, 161) = 4.06, *p* = 0.046, *η*^2^*_p_* = 0.03; originality *F*(1, 161) = 4.81, *p* = 0.030, *η*^2^*_p_* = 0.03), with greater malevolence and originality in the infectious disease prime group (malevolence *M* ± *SD* = 2.12 ± 0.42; originality *M* ± *SD* = 2.09 ± 0.58) than in the natural disaster prime group (malevolence *M* ± *SD* = 1.99 ± 0.43; originality *M* ± *SD* = 1.87 ± 0.58). We did not find any main effect of disease-connoting cue (malevolence *F*(1, 161) = 0.19, *p* = 0.663, *η*^2^*_p_* = 0.001; originality *F*(1, 161) = 0.45, *p* = 0.503, *η*^2^*_p_* < 0.01) or interaction between threat prime and disease-connoting cue (malevolence *F*(1, 161) = 0.05, *p* = 0.826, *η*^2^*_p_* < 0.001; originality *F*(1, 161) = 1.16, *p* = 0.282, *η*^2^*_p_* < 0.01). 

**Figure 3:**
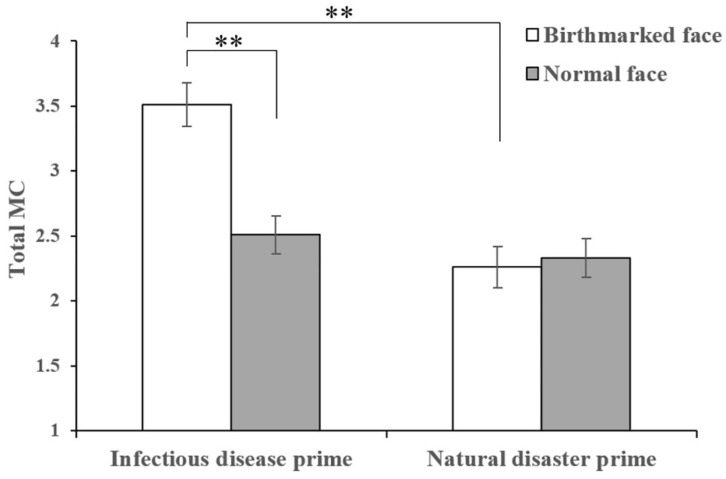
The influence of the interaction between threat prime and disease-connoting cue on total MC (displayed ± *SE*). ** *p* < 0.01.

For the total MC, we did not find a main effect of disease-connoting cue (*F*(1, 161) = 3.15, *p* = 0.078, *η*^2^*_p_* = 0.02). However, we found the main effect of threat prime (*F*(1, 161) = 4.33, *p* = 0.039, *η*^2^*_p_* = 0.03), with greater total MC in the infectious disease prime group (*M* ± *SD* = 3.01 ± 2.09) than in the natural disaster prime group (*M* ± *SD* = 2.29 ± 1.99). Besides, we found a significant interaction between threat prime and disease-connoting cue (*F*(1, 161) = 4.10, *p* = 0.04, *η*^2^*_p_* = 0.03, see Figure 3). For those in the infectious disease prime group, there was a significant effect of disease-connoting cue (*F*(1, 161) = 7.31, *p* = 0.008, *η*^2^*_p_* = 0.04), with greater total MC in the birthmarked face condition than in the normal face condition. For those in the natural disaster prime group, there was no effect of disease-connoting cue (*F*(1, 161) = 0.03, *p* = 0.861, *η*^2^*_p_* < 0.001). Besides, for those in the birthmarked face condition, there was a significant effect of threat prime (*F*(1, 161) = 8.69, *p* = 0.004, *η*^2^*_p_* = 0.05), with greater total MC in the infectious disease prime group than in the natural disaster prime group. However, for those in the normal face condition, there was no significant effect of threat prime (*F*(1, 161) < 0.01, *p* = 0.967, *η*^2^*_p_* < 0.001).

## 3. Study 2

### 3.1. Experimental Design

A 2 (PVD: high, low) × 2 (disease-connoting cue: obese, average-weight) between-subjects design was used. The dependent variables were fluency and originality of malevolent, benevolent, and neutral creativity.

### 3.2. Participants

To select high and low PVD individuals, 533 Chinese college students completed a survey via an online website. The survey includes basic demographic questions and a PVD questionnaire (Duncan et al. 2009). According to their responses to the PVD questionnaire (*M* ± *SD* = 12.74 ± 10.01), we invited 71 high PVD participants and 60 low PVD participants (in the front and last 25% PVD scores in the whole database) to take part in the study. Five participants who doubted the authenticity of the negotiation or the authenticity of two students who might negotiate with participants were excluded from the analyses. One participant was identified as an outlier (more than three standard deviations from the mean for benevolent creativity indicators) and removed from further analyses. The final sample included 125 participants (aged 17 to 28, *M*_age_ = 19.86, *SD*_age_ = 2.11). Among them, 50 (40.0%) were males and the remaining 75 (60.0%) were females.

### 3.3. Materials

#### 3.3.1. Pictures of Obese and Average-Weight Targets

In Study 2, the stimuli of disease-connoting cues were two dieters’ before-and-after photos (one obese and one average-weight pictures in each sex, four pictures in total), which were used in previous research (Lund and Miller 2014; Miller and Maner 2012; Park et al. 2007). To ensure the dieters could be identified as Chinese students, we replaced the heads of dieters with Chinese heads matching sex and weight. Additionally, we used mosaics to cover the Chinese faces considering there might be different attractiveness levels between the faces. Lastly, all the pictures were given discoloration treatments to rule out the color of the target’s clothes as a possible influencing factor.

#### 3.3.2. Negotiation Task

A negotiation task was used to assess participants’ ability of generating malevolent, benevolent, and neutral creativity. Participants were told to take part in a negotiation as the role of either buyer or seller of 50 refrigerators and negotiate a deal with the other person on four issues: price, delivery time, terms of payment, and guarantee (Giebels et al. 1998). Each participant would receive a profit schedule corresponding to his/her role (see Giebels et al. 1998). In the profit schedule, there were possible agreements and the value of those agreements in points. To let the prices in the profit schedule close to the refrigerators’ prices in China, the prices of 50 refrigerators were changed to CNY 200,000/CNY 220,000/CNY 240,000/CNY 260,000/CNY 280,000. Prior issues which had higher values were different between roles. The buyer prioritized delivery time and terms of payment, whereas the seller prioritized price and delivery time. In the case of reaching an agreement, the maximum individual outcome per role was 820, and the minimum individual outcome per role was 0. In case of no agreement, each party would earn zero points. Participants were given 8 min to come up with as many tactics for an effective negotiation as they could think of. Tactics were coded by two trained psychological graduate students who were familiar with the field of creativity and were blind to the conditions and hypotheses. They evaluated non-identical tactics and coded each idea as malevolent, benevolent, or neutral (ICC = 0.82). The discrepancies were solved through discussions. For each participant, their MC, BC, and NC fluency were the number of malevolent, benevolent, and neutral tactics divided by the total number of tactics they generated (De Dreu and Nijstad 2008). Besides, each tactic received an originality score: 1 − (frequency/number of tactics). For example, if a malevolent tactic was mentioned 10 times and all participants generated, together, a total of 100 malevolent tactics, then the tactic received an originality score of 0.9. For each participant, we averaged malevolent tactics’ originality as the original index of malevolent creativity, we averaged benevolent tactics’ originality as the original index of benevolent creativity, we averaged neutral tactics’ originality as the original index of neutral creativity (De Dreu and Nijstad 2008).

#### 3.3.3. Questionnaires

RIBS, MCBS, and PVD were also used in Study 2. The Cronbach’s α coefficient for RIBS, MCBS, and PVD were 0.80, 0.91, and 0.72, respectively.

### 3.4. Procedure

**Figure 4:**
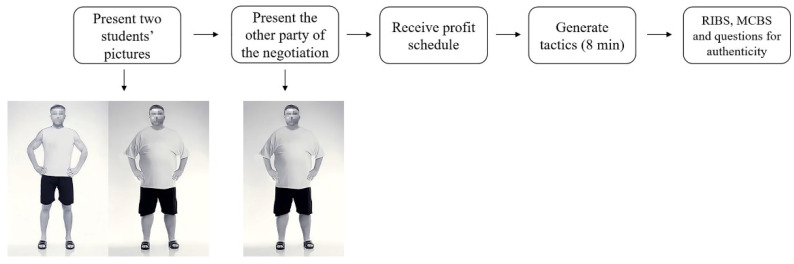
Procedure for male participants in Study 2.

We designed the procedure of Study 2 based on the experimental paradigm used in previous studies, in which high and low PVD participants were selected and instructed to complete a cognitive task that may include disease-connoting cues (Miller and Maner 2012; Park et al. 2007). Specifically, in Study 2, participants were told to take part in the study alone. In the laboratory, they were told that they needed to complete a negotiation task. Then, two students’ pictures were presented on the screen. The two students (one was obese and the other average-weight) were the same sex as the participant. One student’s picture was on the left side of the screen and the other student’s picture was on the right (the locations of the two students’ pictures were counterbalanced between participants). Participants were told that both students were taking part in the same experiment next door and one of the two students would negotiate with them later. Besides, they were told that there were mosaics on the faces of pictures because of privacy issues. Then, the computer randomly selected and then presented one student’s picture as the other party of the negotiation. Next, participants were assigned to take the role of either buyer or seller of 50 refrigerators (the role was counterbalanced). Then, they received their profit schedules. After explaining the content of the schedule, we told them that we were interested in the ways people plan and anticipate negotiations. Therefore, they were given 8 min to first prepare for the upcoming negotiation by generating as many tactics for an effective negotiation as they could think of (De Dreu and Nijstad 2008), typing them into the text-entry box (the picture of obese person or average-weight person was presented on the screen until participants finished the task). After that, participants completed RIBS and MCBS questionnaires. Last, they needed to answer whether they doubted the authenticity of the negotiation or the two students. All the participants received CNY 8 as their compensation. Figure 4 presents the procedure of Study 2.

### 3.5. Result

#### 3.5.1. Effectiveness of Selecting High and Low PVD Participants 

PVD scores were submitted to a 2 (PVD: high, low) × 2 (disease-connoting cue: obese, average-weight) ANOVA. We found the main effect of PVD (*F*(1, 121) = 1233.53, *p* < 0.001, *η*^2^*_p_* = 0.91), with higher scores among high PVD participants (*M* ± *SD* = 26.04 ± 4.64) than among low PVD participants (*M* ± *SD* = −1.21 ± 3.80). Results revealed no main effect of disease-connoting cue (*F*(1, 121) = 0.35, *p* = 0.556, *η*^2^*_p_* < 0.01) and no interaction between PVD and disease-connoting cue (*F*(1, 121) = 0.11, *p* = 0.742, *η*^2^*_p_* = 0.001). These results showed that we successfully selected high and low PVD participants.

#### 3.5.2. Creativity

Means and standard deviations of malevolent, benevolent, and neutral creativity in Study 2 are presented in Table 3.

We submitted fluency and originality of malevolent, benevolent, and neutral creativity to separate 2 (PVD: high, low) × 2 (disease-connoting cue: obese, average-weight) ANOVAs with RIBS and MCBS as covariates. 

**Figure 5:**
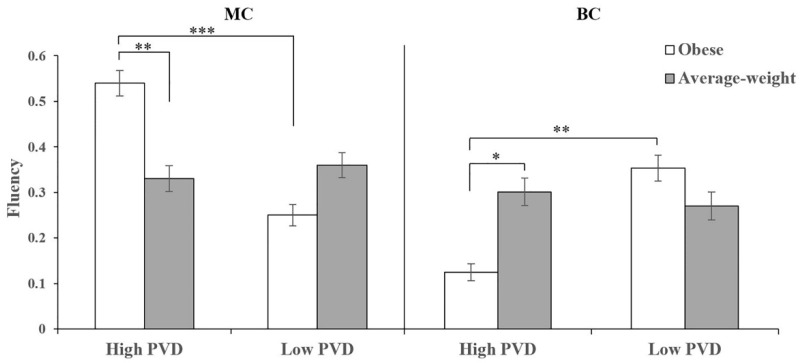
The influences of the interaction between PVD and disease-connoting cue on MC fluency and BC fluency (displayed ± *SE*). * *p* < 0.05; ** *p* < 0.01; *** *p* < 0.001.

**Figure 6:**
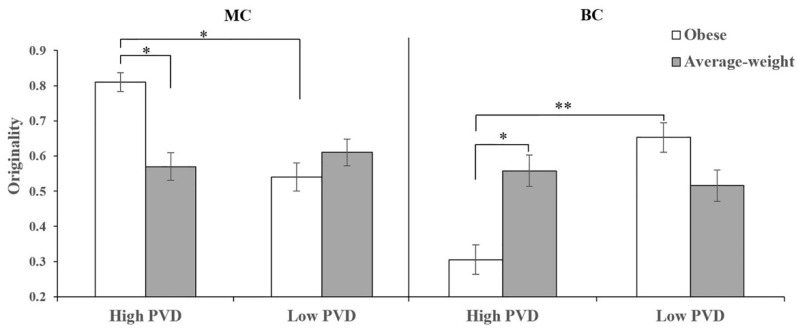
The influences of the interaction between PVD and disease-connoting cue on MC originality and BC originality (displayed ± *SE*). * *p* < 0.05; ** *p* < 0.01.

For the MC fluency, we found a main effect of PVD (*F*(1, 119) = 5.50, *p* = 0.021, *η*^2^*_p_* = 0.04), with higher fluency among high PVD individuals (*M* ± *SD* = 0.43 ± 0.33) than among low PVD individuals (*M* ± *SD* = 0.31 ± 0.29). Although we did not find a main effect of disease-connoting cue (*F*(1, 119) = 0.81, *p* = 0.369, *η*^2^*_p_* < 0.01), we found a significant interaction between PVD and disease-connoting cue (*F*(1, 119) = 8.60, *p* = 0.004, *η*^2^*_p_* = 0.07, see Figure 5). As expected given our results, among high PVD participants there was a significant effect of disease-connoting cue (*F*(1, 119) = 8.27, *p* = 0.005, *η*^2^*_p_* = 0.07), with higher fluency in the obese condition than in the average-weight condition. Among low PVD participants, there was no significant effect of disease-connoting cue (*F*(1, 119) = 1.88, *p* = 0.173, *η*^2^*_p_* = 0.02). Besides, for those in the obese condition, there was a significant effect of PVD (*F*(1, 119) = 13.85, *p* < 0.001, *η*^2^*_p_* = 0.10), with higher fluency among high PVD participants than among low PVD participants. However, for those in the average-weight condition, there was no significant effect of PVD (*F*(1, 119) = 0.18, *p* = 0.674, *η*^2^*_p_* = 0.001); for the MC originality, we did not find any main effect (PVD *F*(1, 119) = 2.04, *p* = 0.156, *η*^2^*_p_* = 0.02; disease-connoting cue *F*(1, 119) = 1.27, *p* = 0.261, *η*^2^*_p_* = 0.01). However, we found a significant interaction effect (*F*(1, 119) = 4.49, *p* = 0.036, *η*^2^*_p_* = 0.04, see Figure 6). Among high PVD participants, there was a significant effect of disease-connoting cue (*F*(1, 119) = 5.93, *p* = 0.016, *η*^2^*_p_* = 0.05), with higher originality in the obese condition than in the average-weight condition. Among low PVD participants, there was no significant effect of disease-connoting cue (*F*(1, 119) = 0.45, *p* = 0.503, *η*^2^*_p_* < 0.01). Besides, for those in the obese condition, there was a significant effect of PVD (*F*(1, 119) = 6.26, *p* = 0.014, *η*^2^*_p_* = 0.05), with higher originality among high PVD participants than among low PVD participants. However, for those in the average-weight condition, there was no significant effect of PVD (*F*(1, 119) = 0.24, *p* = 0.622, *η*^2^*_p_* < 0.01).

For the BC fluency and originality, we did not find any main effect (*ps* ≥ 0.077). However, we found significant interaction effects on both fluency (*F*(1, 119) = 5.20, *p* = 0.024, *η*^2^*_p_* = 0.04, see Figure 5) and originality (*F*(1, 119) = 5.00, *p* = 0.027, *η*^2^*_p_* = 0.04, see Figure 6). Among high PVD individuals, there were significant effects of disease-connoting cue (fluency *F*(1, 119) = 5.28, *p* = 0.023, *η*^2^*_p_* = 0.04; originality *F*(1, 119) = 4.55, *p* = 0.035, *η*^2^*_p_* = 0.04), with lower fluency and originality in the obese condition than in the average-weight condition. Among low PVD individuals, the effects of disease-connoting cue were non-significant (fluency *F*(1, 119) = 1.02, *p* = 0.314, *η*^2^*_p_* < 0.01; originality *F*(1, 119) = 1.21, *p* = 0.274, *η*^2^*_p_* = 0.01). Besides, for those in the obese condition, there were significant effects of PVD (fluency *F*(1, 119) = 8.23, *p* = 0.005, *η*^2^*_p_* = 0.07; originality *F*(1, 119) = 7.85, *p* = 0.006, *η*^2^*_p_* = 0.06), with lower fluency and originality among high PVD participants than among low PVD participants. However, for those in the average-weight condition, the effects of PVD were non-significant (fluency *F*(1, 119) = 0.13, *p* = 0.723, *η*^2^*_p_* = 0.001; originality *F*(1, 119) = 0.13, *p* = 0.720, *η*^2^*_p_* = 0.001).

For the NC fluency and originality, we did not find any significant main effect (*ps* ≥ 0.141) or interaction effect (*ps* ≥ 0.525).

## 4. General Discussion

The current article investigated the effect of infectious disease threat on MC by two disease-connoting cues (birthmark, obesity). Across two studies, we found that disease-connoting cues induced MC among people whose BIS was situationally (Study 1) or chronically (Study 2) activated. Specifically, in Study 1, we found that, compared with the natural disaster prime group, the infectious disease prime group showed higher MC fluency and total MC when they were provoked by a birthmark person. Compared with participants in the normal face condition, participants in the birthmarked face condition showed higher MC fluency and total MC when they were exposed to infectious disease prime. In Study 2, we found that, compared with low PVD participants, high PVD participants showed higher MC fluency and originality when they negotiated with an obese person. Compared with the average-weight condition, high PVD participants in the obese condition showed higher MC fluency and originality. In summary, our results indicated that infectious disease threats could increase MC.

Results of Study 1 found that, compared with participants in the normal face condition, participants in the birthmarked face condition showed higher MC fluency and total MC when they were exposed to situational infectious disease prime. According to the “Functional Flexibility Principle” (Schaller and Park 2011; Schaller et al. 2007), when situational disease prime activates people’s BIS, disease-connoting cues could make people feel threatened by infectious disease and induce their malevolence. Indeed, previous studies found that people who were exposed to disease prime linked disease-connoting cues (e.g., obesity) with the presence of disease (Duncan and Schaller 2009; Park et al. 2007) and showed higher levels of malevolence (e.g., prejudice, exclusion) towards individuals with disease-connoting cues (Huang et al. 2011; Lund and Miller 2014; Makhanova et al. 2015; Wu et al. 2019). Therefore, in this study, when infectious disease video activated participants’ BIS, a birthmarked face rather than a normal face might make participants feel threatened by infectious disease, which further led to higher malevolence. Malevolence is a necessary feature of MC. According to the motivated focus account of creativity (De Dreu and Nijstad 2008), the threat could promote creativity when creativity is relevant to the threat. Therefore, in Study 1, compared with a normal face, a birthmarked face could increase MC when participants were exposed to situational infectious disease prime. Besides, we found that when participants were primed with natural disaster, there was no significant difference between participants in the birthmarked face condition and participants in the normal face condition. Because a natural disaster video can’t activate a human’s BIS in theory, a birthmarked face may not make participants in the natural disaster prime group feel threatened by infectious disease, which leads to low MC towards a birthmarked face. This result also indicated that birthmarks could make people feel threatened by infectious diseases and increase people’s MC only when their BIS was activated (e.g., primed with an infectious disease rather than other kinds of threats).

Results of Study 2 showed that, compared with average weight, obesity induced a higher MC fluency and originality among high PVD participants. According to the “Functional Flexibility Principle” (Schaller and Park 2011; Schaller et al. 2007), when chronically feeling vulnerable to disease (e.g., high in PVD) activates people’s BIS, people might associate disease-connoting cues (e.g., obesity, birthmark) with infectious disease. Therefore, in this study, among high PVD participants whose BIS might be activated, an obese figure rather than an average-weight figure might make them feel threatened by infectious disease and induce their malevolence, which further leads to the generation of MC. Besides, the results of Study 2 showed there was no significant difference between obese condition and average-weight condition among low PVD participants, which might be due to the low PVD trait that couldn’t activate people’s BIS. According to the “Functional Flexibility Principle” (Schaller and Park 2011; Schaller et al. 2007), in the absence of situational disease prime, low PVD individuals will not overact to individuals with disease-connoting cues (e.g., obese people), which may lead them to solve the problem equally with an average-weight person and obese person. This result also indicated that obesity could make people feel threatened by infectious diseases and increase people’s MC only when their BIS was activated (e.g., high PVD trait). Different from MCT in which participants are instructed to complete a malevolent-oriented task, the negotiation task is not framed to be malevolent, and participants in the negotiation task are allowed to generate MC, BC, or NC freely. Therefore, Study 2 extended the conclusion of Study 1 by indicating that infectious disease threats could increase MC even in situations that were not emotionally laden. 

Results of Study 2 also showed that, compared with average weight, obesity induced a lower BC fluency and originality among high PVD participants. Besides, compared with low PVD participants, high PVD participants showed lower BC fluency and originality when they negotiated with an obese person. Previous studies suggested that, among people who were chronically feeling vulnerable to disease (e.g., high in PVD), disease-connoting cues (e.g., obesity) could make them feel threatened by infectious disease and increase their malevolent attitudes as well as malevolent behaviors, such as aggression, prejudice, and exclusion (Loustau 2021; Magallares et al. 2015; Makhanova et al. 2015; Park et al. 2007). Benevolence is an essential prerequisite of BC. Although infectious disease threats could mobilize cognitive resources and increase MC, the increased malevolence might decrease benevolence and BC in this study. 

## 5. Future Directions

There are several problems to solve in the future. First, creativity includes not only idea generation but also idea evaluation and selection (Rietzschel et al. 2006). Thus, future studies are needed to explore the effect of disease threat on MC evaluation and selection. Secondly, future studies need to examine whether disease protections, such as washing hands and vaccination, could alleviate the effects of disease threats on MC. Lastly, the neural mechanism of the effect of infectious disease threat on MC needs to be explored.

## 6. Conclusions

In summary, infectious disease threats could promote the idea generation of MC. The results suggest that people’s malevolent creativity, as well as destructiveness, might be induced by disease-connoting cues during the pandemic, and necessary interventions are needed.

## Figures and Tables

**Table 1:** Means and standard deviations of manipulation checks.

	Infectious Disease Prime Group	Natural Disaster Prime Group
Happy	1.28 (0.71)	1.34 (0.65)
Sad	5.35 (1.49)	5.54 (1.50)
Tense	6.05 (1.06)	6.07 (1.14)
Surprise	4.21 (1.63)	3.96 (1.66)
Disgust	4.78 (1.84)	2.83 (1.32)
Fear	5.52 (1.39)	5.83 (1.26)
Perceived threat level	5.26 (1.58)	5.23 (1.65)
Disease content relevance	6.34 (1.00)	1.70 (1.02)
Natural disaster content relevance	2.47 (1.37)	6.70 (0.56)
Disease concerns	6.22 (0.75)	2.10 (1.20)
Natural disaster concerns	3.22 (1.62)	6.35 (0.68)

Note: Standard deviation in parentheses.

**Table 2:** Means and standard deviations of MC in Study 1.

	Infectious Disease Prime Group		Natural Disaster Prime Group	
	Birthmarked Face	Normal Face	Birthmarked Face	Normal Face
Fluency	5.16 (2.15)	4.18 (2.03)	4.14 (2.16)	4.41 (2.07)
Malevolence	2.14 (0.35)	2.11 (0.48)	2.01 (0.43)	1.97 (0.44)
Originality	2.14 (0.51)	2.04 (0.64)	1.85 (0.53)	1.89 (0.64)
Total MC	3.51 (2.16)	2.51 (1.91)	2.26 (2.06)	2.33 (1.94)

Note: Standard deviation in parentheses.

**Table 3:** Means and standard deviations of malevolent, benevolent, and neutral creativity in Study 2.

		High PVD Participants		Low PVD Participants	
		Obese	Average-Weight	Obese	Average-Weight
MC	Fluency	0.54 (0.31)	0.33 (0.32)	0.25 (0.26)	0.36 (0.31)
	Originality	0.81 (0.30)	0.57 (0.44)	0.54 (0.45)	0.61 (0.43)
BC	Fluency	0.12 (0.20)	0.29 (0.32)	0.34 (0.31)	0.26 (0.33)
	Originality	0.30 (0.45)	0.54 (0.47)	0.63 (0.44)	0.50 (0.47)
NC	Fluency	0.34 (0.34)	0.39 (0.36)	0.41 (0.36)	0.38 (0.29)
	Originality	0.60 (0.43)	0.56 (0.40)	0.73 (0.40)	0.64 (0.36)

Note: Standard deviation in parentheses.

## Data Availability

The data are currently not publicly available due to participant privacy, but they are available from the corresponding author upon reasonable request.
